# A novel approach to rapidly prevent age-related cognitive decline

**DOI:** 10.1111/acel.12178

**Published:** 2013-12-04

**Authors:** Paul A Adlard, Amelia Sedjahtera, Lydia Gunawan, Lisa Bray, Dominic Hare, Jessica Lear, Philip Doble, Ashley I Bush, David I Finkelstein, Robert A Cherny

**Affiliations:** 1 The Florey Institute of Neuroscience and Mental Health, Kenneth Myer Building, At Genetics Lane on Royal Parade, The University of MelbourneMelbourne, Vic., 3010, Australia; 2 Elemental Bio-imaging Facility, University of TechnologySydney, NSW, 2007, Australia

**Keywords:** aging, anti-aging, cognition, PBT2, zinc

## Abstract

The loss of cognitive function is a pervasive and often debilitating feature of the aging process for which there are no effective therapeutics. We hypothesized that a novel metal chaperone (PBT2; Prana Biotechnology, Parkville, Victoria, Australia) would enhance cognition in aged rodents. We show here that PBT2 rapidly improves the performance of aged C57Bl/6 mice in the Morris water maze, concomitant with increases in dendritic spine density, hippocampal neuron number and markers of neurogenesis. There were also increased levels of specific glutamate receptors (alpha-amino-3-hydroxy-5-methyl-4-isoxazolepropionic acid and *N*-methyl-d-aspartate), the glutamate transporter (VGLUT1) and glutamate itself. Markers of synaptic plasticity [calmodulin-dependent protein kinase II (CaMKII) and phosphorylated CaMKII, CREB, synaptophysin] were also increased following PBT2 treatment. We also demonstrate that PBT2 treatment results in a subregion-specific increase in hippocampal zinc, which is increasingly recognized as a potent neuromodulator. These data demonstrate that metal chaperones are a novel approach to the treatment of age-related cognitive decline.

## Introduction

The normal aging process is characterized by a decline in function across multiple physiological domains, including cognition. These deficits are distinguished from pathological aging processes such as Alzheimer’s disease (AD), by the rate and the magnitude of the deficits. While there have been a number of reports on the prevalence of age-related cognitive decline (ARCD), a recent longitudinal study in the USA (Plassman *et al*., 2008) reported that in 2002, there were an estimated 5.4 million people (22.2%) aged 71 years and older that had cognitive impairment without dementia, suggesting that ARCD may be more prevalent than dementia itself (Plassman *et al*., 2007). While aging does not affect all cortical regions or cognitive domains equally, there is evidence that memory performance does undergo a conspicuous decline with aging. Specifically, delayed recall of verbal information (Albert *et al*., 1987), working memory, short-term recall (Craik *et al*., 1994) and spatial memory (Montgomery *et al*., 2000) all show a gradual decline across age in the human population. Cumulatively, these memory impairments tangibly impact an individual’s activities of daily living.

There are currently no therapeutics that effectively target the symptoms, let alone the biological substrates, of ARCD. Given the potential symptomatic and mechanistic overlap between aging and AD, we hypothesized that a metal chaperone (PBT2; Prana Biotechnology) previously shown to benefit synaptic plasticity-related endpoints and/or cognitive function in transgenic mouse models of AD (Adlard *et al*., 2008, 2011) and in human clinical trials for patients with mild AD (Lannfelt *et al*., 2008; Faux *et al*., 2010) would enhance cognition in aged mice. We show here that PBT2 improves learning and memory in aged wild-type mice, concomitant with alterations to anatomical and biochemical substrates that are critical to normal cognitive function and which suggest a specific effect of PBT2 on enhancing glutamatergic signalling pathways at the synapse. These data demonstrate that metal chaperones are a novel therapeutic approach for the treatment of ARCD. In addition, we have shown a PBT2-mediated modulation of several cellular targets relevant to other neurodegenerative disorders, further highlighting the potential benefit of PBT2.

## Results

### PBT2 restores the performance of aged mice on the Morris Water maze

**Figure 1:**
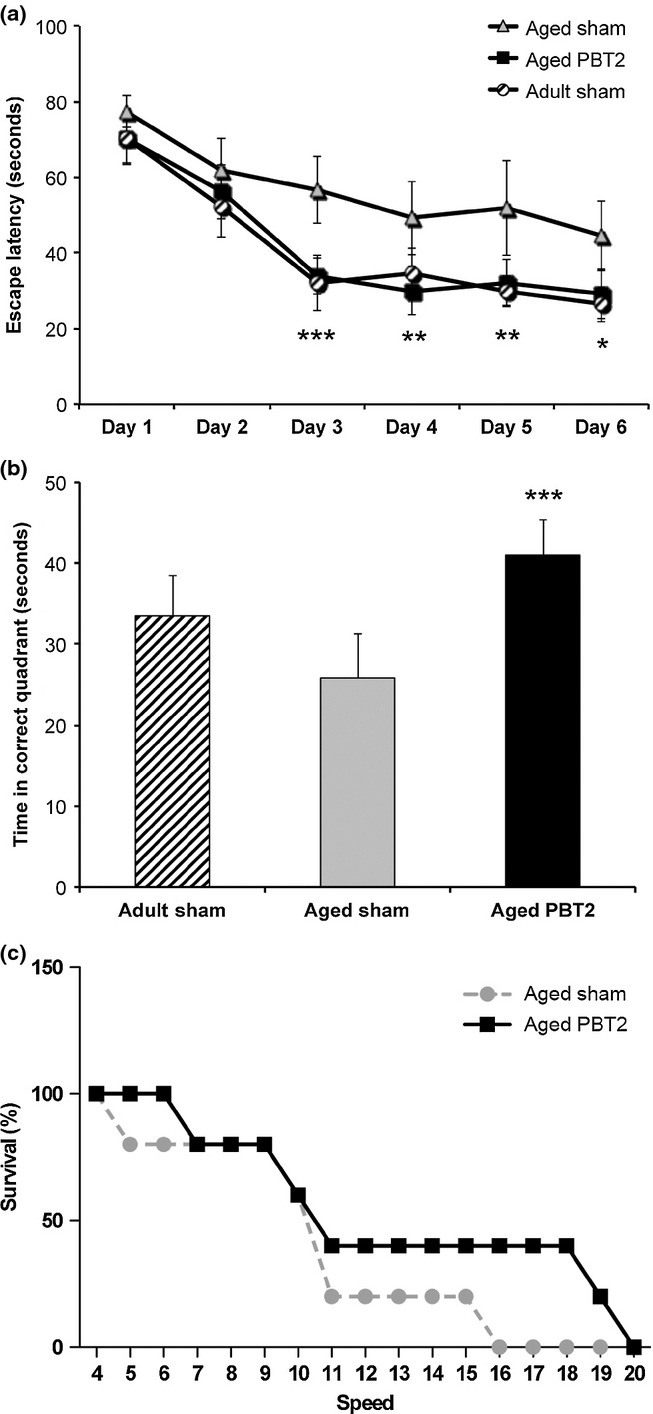
PBT2 improves behavioural outcomes in aged mice. (a) Performance in the learning component of the Morris water maze was assessed over six consecutive days, with mice receiving four trials per day. Data shown are the average escape latencies (seconds). Aged mice (*n* = 21) were significantly impaired as compared to adult sham-treated animals (*n* = 7). Acute PBT2 treatment of aged mice (*n* = 23) resulted in a significant improvement in performance across this task (*P* = 0.004), with significant differences between the groups emerging on day 3 of testing and persisting for the remainder of the testing period. (b) Performance in the recall task, conducted 24 h after the final learning trial, demonstrated that PBT2 significantly improved the performance of the aged mice (*P* = 0.0005), which were indistinguishable from adult control animals. (c) Rotarod data, showing the percentage survival of mice on the rod at the different speeds. The PBT2-treated mice (*n* = 5) performed significantly better (*P* < 0.05) on this task than the sham-treated controls (*n* = 5). All data are averages ± SEM. *P < 0.05, **P < 0.01, ***P < 0.001.

Aged wild-type mice (22 months) were treated for a total period of 12 days (30 mg/kg/day PBT2 or vehicle), during which time their performance on the Morris water maze was assessed, and compared to a cohort of vehicle-treated adult wild-type mice (12 months). There was a significant overall effect of time (two-way ANOVA, *P* < 0.0001) and treatment (*P* = 0.0025) in this task. Aged mice were impaired in the acquisition of this spatial memory task (repeated-measures ANOVA, *P* = 0.004) (Fig. 1a) and showed a trend to a decreased recall of the task (Fig. 1b). This age-related impairment in spatial memory is conserved across multiple species, including monkeys (Lai *et al*., 1995) and mice (Bach *et al*., 1999). In our paradigm, aged mice treated with PBT2 showed a significant improvement in performance in both the acquisition (day 3, *P* = 0.0009, day 4, *P* = 0.004, day 5, *P* = 0.006, day 6, *P* = 0.01) and the recall (*P* = 0.0005) of the spatial memory task compared to aged vehicle-treated mice (there was no effect of PBT2 on swim speed) and were indistinguishable from adult animals (Fig. 1a,b). In a separate group of mice, PBT2 did not significantly affect exploratory behaviour in the open field test (distance moved, number of moves, time in movement or velocity; data not shown) or motor coordination in the pole test (time to turn or time to complete; data not shown), but did produce a small significant benefit [log-rank (Mantel–Cox) test, *P* = 0.01] on gross motor performance in the accelerating rotarod [survival (%) on the rod, Fig. 1c; there was no effect of PBT2 on latency to fall on the rotarod (data not shown)]. These data suggest that PBT2 functions as a cognitive enhancer to prevent ARCD.

### PBT2 increases dendritic spine density

In order to provide insight into the potential mechanisms of action of PBT2, we examined several physiological parameters related to learning and memory/cognition that we have previously shown to be altered following PBT2 treatment in transgenic mouse models of AD. A number of these ‘biomarkers’ may be relevant to both normal and pathological aging, and other diseases or conditions that are not necessarily age dependent.

**Figure 2:**
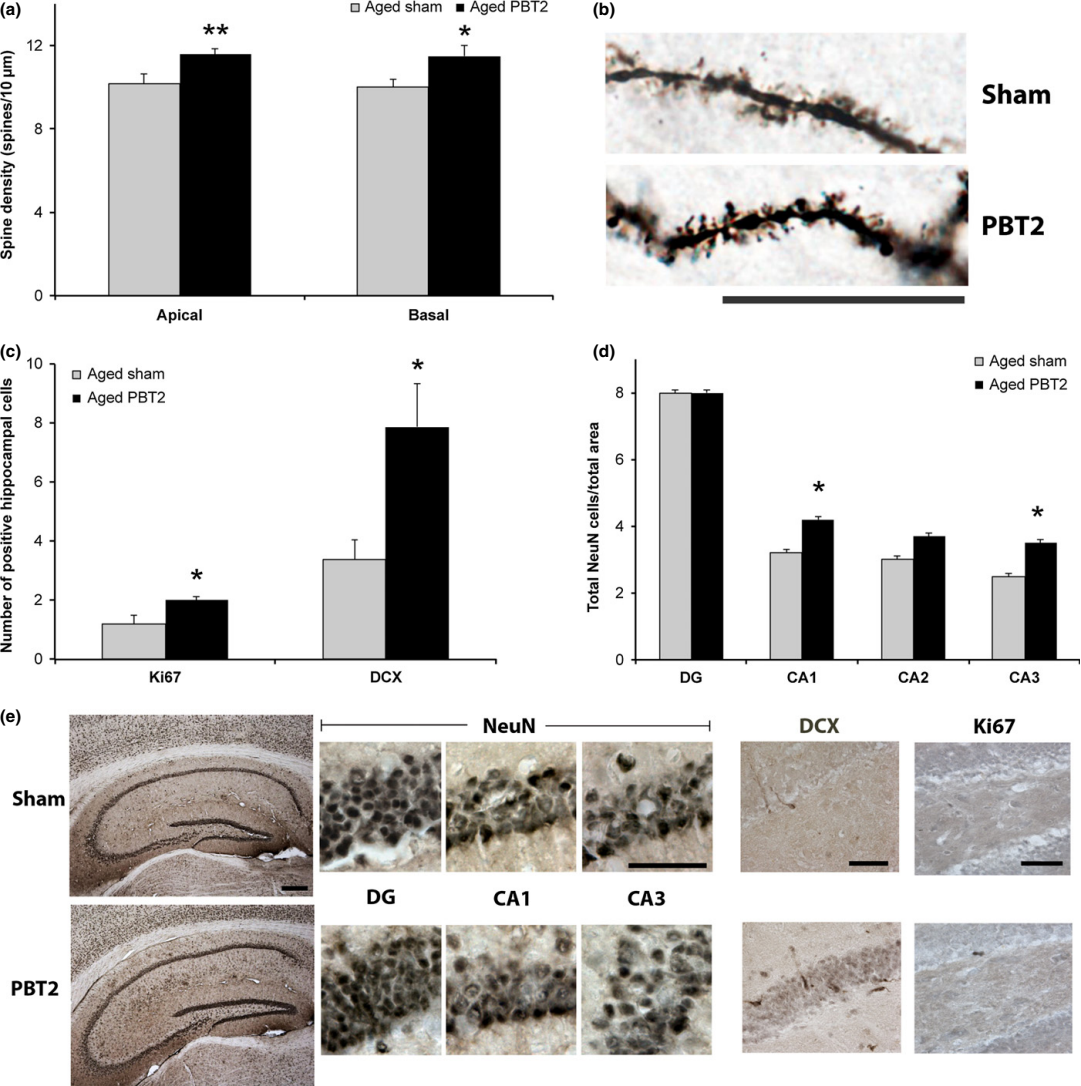
PBT2 improves histological endpoints in aged mice. (a) Golgi analysis was conducted on aged mice (50 neurites per mouse, *n* = 6 PBT2, *n* = 5 sham), with PBT2 treatment resulting in a significant increase in both apical and basal spine density. Young control mice (4 months of age, 50 neurites per mouse, *n* = 3, data not shown) were also analysed, revealing spine density values of 10.4 ± 1.1 (basal) and 12.8 ± 1.3 (apical). PBT2 did not significantly alter apical or basal spine density in young control mice [4 months of age, 50 neurites per mouse, *n* = 3, data not shown; 10.15 ± 1.3 (basal) and 12.15 ± 1.5 (apical)]. (b) A sample golgi image. Scale bar = 25 μm. (c) Stereological quantitation of the number of Ki67- and doublecortin (DCX)-positive cells in the hippocampus revealed small but significant increases in both following PBT2 treatment (*n* = 4–6 mice per group with *n* = 4 sections per mouse). Ki67-positive cells were also significantly increased surrounding the dorsal third ventricle following PBT2 treatment (+214%) in aged animals [*n* = 5–7 mice per group with *n* = 4 sections per mouse, *P* < 0.001 (data not shown)]. (d) Stereological quantitation of the number of NeuN-positive neurons in the various hippocampal subfields revealed a significant increase in neurons in area CA1 and CA3 following PBT2 treatment (*n* = 4 mice per group with *n* = 4 sections per mouse). (e) Sample histological images. The first panel shows the entire hippocampus stained for NeuN (scale bar = 250 μm), while the remaining panels show higher power images of NeuN, DCX and Ki67 staining (scale bars = 50 μm). All data are averages ± SEM. *P < 0.05, **P < 0.01, ***P < 0.001.

Using Golgi impregnation, we examined the effect of PBT2 on dendritic spine density and dendritic length in the hippocampus. Dendritic spines are dynamic anatomical structures believed critical for learning and memory, which are rapidly modulated in response to various stimuli and are the site of the majority of postsynaptic excitatory glutamatergic synapses in the hippocampus. The molecular constituents of spines, and the associated dendritic length/branching to a lesser extent, may promote functional plasticity and/or be modulated in both normal and pathological aging. While PBT2 did not result in a statistically significant change in dendritic length [basal: PBT2 (+14%), *P* > 0.05; apical: PBT2 (+5%), *P* > 0.5], there was a significant increase in spine density on the basal and apical dendrites [basal: PBT2 (+15%), *P* = 0.02; apical: PBT2 (+14%), *P* = 0.009] (Fig. 2a,b). Such anatomical alterations (increased spine density in the absence of any change in dendritic length) have also been observed following stimuli, such as environmental enrichment, which also enhances spatial memory (Moser *et al*., 1997). Thus, PBT2 may be modulating the activity/plasticity of glutamatergic synapses to affect the improved learning and memory observed in the aged mice. Mechanistically, this may relate to an effect on long-term potentiation (LTP), whose induction and maintenance is impaired in the aged hippocampus (Barnes, 1979; Barnes *et al*., 2000) and whose magnitude is greatest in basal dendrites.

### PBT2 increases markers of neurogenesis and neuron number

While the loss of synaptic connectivity in the context of normal aging is generally considered to be a mediator of cognitive decline, as opposed to overt neuron loss, we assessed the markers of both neurogenesis and neuron number in the hippocampus, as this may contribute to the function of this brain region, as well as limit subsequent susceptibility to degeneration resulting from the onset of neurological disease. A histological examination of surrogate markers for neurogenesis revealed that PBT2 significantly increased the number of Ki-67-positive cells [PBT2 (+67%), *P* = 0.05], a proliferation marker, and doublecortin-positive cells [PBT2 (+130%), *P* = 0.01], a marker for immature neurons, in the hippocampus (Fig. 2c,e). Ki67-positive cells were also significantly increased [PBT2 (+214%), *P* = 0.008] surrounding the dorsal third ventricle following PBT2 treatment (*n* = 5–7 per group, four sections per group; data not shown). Further examination of the number of cells labelled for NeuN, a marker for mature neurons, also revealed a significant increase in neurons in area CA1 [PBT2 (+27%), *P* = 0.01] and CA3 [PBT2 (+21%), *P* = 0.04] of the hippocampus following PBT2 treatment (Fig. 2d,e). These data suggest that PBT2 may foster a brain microenvironment that is conducive to neuronal health, which may help overcome age- or disease-related losses in neurogenesis (Lazarov *et al*., 2010) and cell death (Stranahan *et al*., 2012) and ultimately cognition.

### PBT2 causes a subregion-specific increase in zinc in the hippocampus

The PBT2-mediated functional improvements are likely to be supported by both anatomical and biochemical alterations. As we have previously hypothesized that the principal mechanism of action of PBT2 is to modulate metal levels (particularly zinc) and we have also shown that synaptic zinc is crucial for the maintenance of normal cognition (Adlard *et al*., 2010), we examined zinc distribution within the hippocampus as a primary upstream biochemical mediator of the cognitive benefit observed in this study.

**Figure 3:**
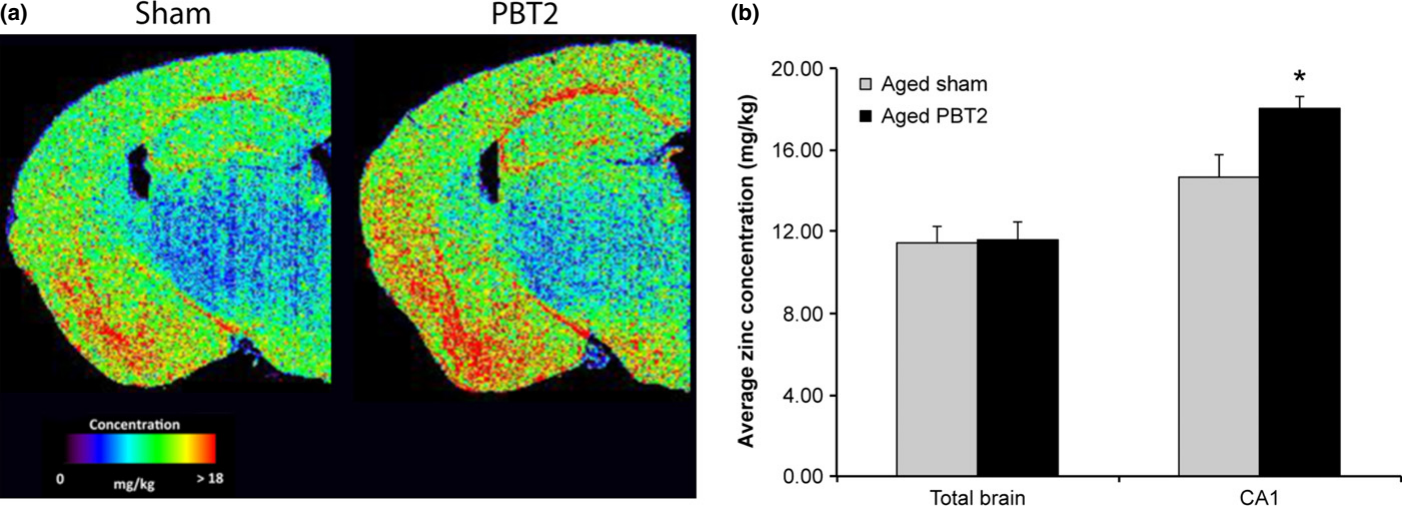
PBT2 modulates zinc levels in the hippocampus. (a) A sample laser ablation inductively coupled plasma mass spectrometry (ICPMS) image showing the distribution of zinc in the aged mouse brain, both with and without PBT2 treatment. (b) Quantitation of the laser ablation ICPMS data showing that PBT2 treatment gives rise to an increased level of zinc in area CA1 of the hippocampus, but overall total brain levels are unchanged at this resolution (*n* = 5–6 per group).

Utilizing laser ablation inductively coupled plasma mass spectrometry (LA-ICPMS), we assessed average concentrations of zinc within the entire brain, cortex, amygdala and the various hippocampal subfields. These data revealed that the average zinc concentration across the entire brain in the aged animals was unchanged following PBT2 treatment (Fig. 3a,b), consistent with solution ICPMS of brain homogenates (data not shown). There was, however, a trend to an increase in the average zinc concentration within the cortex, hippocampus and amygdala of PBT2-treated aged mice, an increase that diminished the difference in zinc levels seen between adult and aged mice (Fig. S1, Supporting Information). A finer delineation of zinc concentrations within the hippocampal subfields revealed that there was a significant increase [PBT2 (+23%), *P* = 0.04] in zinc in area CA1 of the hippocampus (Fig. 3a,b) [average copper concentrations in the total brain as well as in the isolated hippocampus, cortex and amygdala were unchanged following PBT2 treatment (Fig. S1) and subregion analysis of the hippocampus did not reveal any significant effect of PBT2 on modulating copper levels (data not shown)]. This is relevant as zinc, which is co-released in high concentration at the synapse during activity (Frederickson *et al*., 2006) together with glutamate, plays a crucial role in learning and memory via its function as a neuronal messenger and a modulator of synaptic transmission and plasticity (Smart *et al*., 2004; Paoletti *et al*., 2009). Furthermore, an increase in CA1 levels of zinc may specifically aid in the restoration/maintenance of LTP (Takeda *et al*., 2009).

### PBT2 increases VGLUT1 and glutamate levels

**Figure 4:**
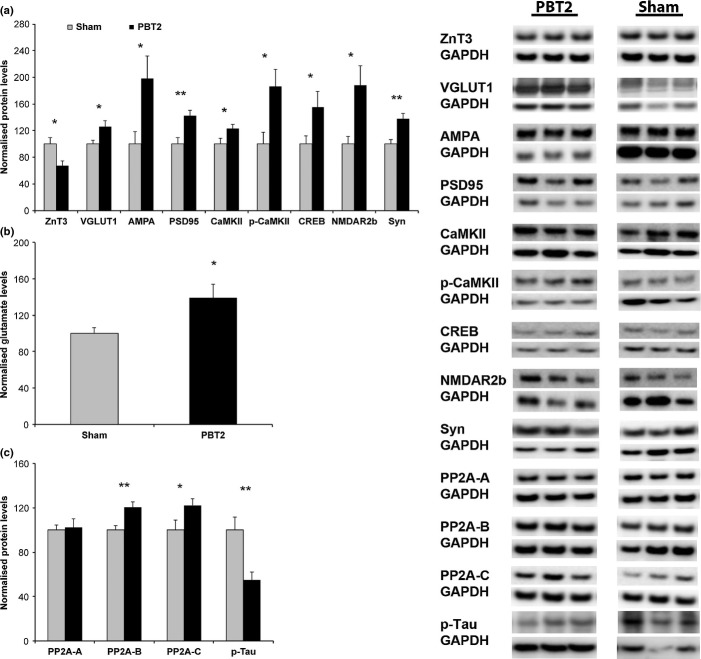
PBT2 modulates biochemical endpoints in the hippocampus. (a) Quantitation of Western blot data (sample composite images shown on the right). The synaptic vesicle zinc transporter, ZnT3, was decreased in the PBT2-treated mice as compared to sham-treated animals. In contrast, the glutamate transporter, VGLUT1, was significantly increased following PBT2 treatment, as compared to sham-treated controls. The levels of other synaptic proteins involved in cognition and plasticity – AMPA and NMDA, PSD95, CREB, CaMKII, phosphorylated CaMKII and synaptophysin (Syn) – were also increased following PBT2 treatment of the aged mice (*n* = 9–15 per group). (b) Total glutamate levels were also significantly increased in the aged mice following PBT2 treatment (*n* = 10 per group). (c) The levels of the different protein phosphatase 2A subunits (A, B, C) were also altered following PBT2 treatment (*n* = 12 per group), as was one of its primary substrates, phosphorylated tau (*n* = 8 per group) (sample composite images shown on the right). For all Western blot results, data were normalized to GAPDH and then presented as normalized to sham-treated values (100%). All data are averages ± SEM. *P < 0.05, **P < 0.01.

Whether the increase in cellular zinc levels directly translates to an increased availability of zinc specifically at the synapse is unclear. To this end, we examined the levels of the zinc transporter 3 (ZnT3) protein in the hippocampus. ZnT3 is primarily localized to glutamatergic synapses (Palmiter *et al*., 1996) and is essential for loading zinc into synaptic vesicles (Cole *et al*., 1999). We have previously shown that the ablation of ZnT3 results in a profound age-dependent cognitive phenotype and that there is an age-related decline in this protein in both normal mice and healthy older adults and a further exaggerated decline in AD (Adlard *et al*., 2010). Thus, in addition to an increase in zinc levels, a plausible mechanistic pathway to account for the restored cognitive function following PBT2 treatment observed in this study would require an upregulation of a synaptic vesicle zinc transporter such as ZnT3 (PBT2 itself may also be acting as a surrogate vesicular/synaptic zinc transporter, which is the subject of ongoing investigations). This would ensure the correct localization of zinc that would then facilitate the cellular signalling pathways required for cognition. Surprisingly however, there was a significant decrease in total hippocampal ZnT3 levels following PBT2 treatment [PBT2 (−33%), *P* = 0.01] (Fig. 4a), suggesting that another zinc transporter may be activated following PBT2 treatment. Thus, we examined the levels of the vesicular glutamate transporter 1 (VGLUT1) in the hippocampus.

VGLUT1 primarily functions to regulate the packaging of l-glutamate into synaptic vesicles within glutamatergic neurons in the hippocampus and other select areas of the brain (Takamori, 2006), impacting glutamate quantal size and synaptic glutamate release (Wilson *et al*., 2005), but has also been shown to increase vesicular zinc uptake (and may be the rate-limiting step in zinc transport) (Salazar *et al*., 2005), and it coexists in the same vesicle as ZnT3. VGLUT1 was significantly increased following PBT2 treatment [PBT2 (+26%), *P* = 0.03] (Fig. 4a), and this was concomitant with a significant net increase in glutamate levels in the PBT2-treated mice [PBT2 (+39%), *P* = 0.02] (Fig. 4b). While this implies the potential for an effect on synaptic zinc content, it clearly provides further evidence for a positive benefit of PBT2 on cognition, as increased VGLUT1 and glutamate levels may impact synaptic transmission and serve as an indicator of relative synaptic strength of presynaptic glutamatergic innervation (Wojcik *et al*., 2004). Furthermore, VGLUT1 is tightly correlated with cognitive performance in humans (Kashani *et al*., 2008). Such changes are likely to oppose the age-related decreases that have been reported for both VGLUT1 and glutamate (Minkeviciene *et al*., 2008; Chang *et al*., 2009).

### PBT2 increases glutamate receptors and markers of synaptic plasticity in the hippocampus

To further interrogate the glutamate pathway, we examined the levels of the ionotropic glutamate receptors, the alpha-amino-3-hydroxy-5-methyl-4-isoxazolepropionic acid (AMPA) and *N*-methyl-d-aspartate (NMDA) receptors and found both to be increased following PBT2 treatment [AMPA: PBT2 (+97%), *P* = 0.03; NMDAR2b: PBT2 (+88%), *P* = 0.03; Fig 4a] (levels of NMDAR1 and NMDAR2a were not significantly altered following PBT2 treatment, data not shown). Other biochemical changes included increases in postsynaptic density 95 [PBT2 (+42%), *P* = 0.004], cAMP response element-binding protein [PBT2 (+55%), *P* = 0.04], calcium/calmodulin-dependent protein kinase II (CaMKII) [PBT2 (+23%), *P* = 0.04] and phosphorylated CaMKII [PBT2 (+86%), *P* = 0.02)] and synaptophysin [PBT2 (+38%), *P* = 0.008] (Fig. 4a). This series of proteins are functionally intertwined at the synapse and are critically involved in cognitive function and synaptic plasticity (Lee & Silva, 2009; Lisman *et al*., 2012). These data demonstrate that the cognitive benefits of PBT2 are likely to be at least in part mediated by an action on glutamatergic neurons/synapses.

### PBT2 increases protein phosphatase 2A

Our previous publications have clearly shown that PBT2 has a breadth of biological activities that may also contribute, either directly or indirectly, to an enhancement of cognition. Most recently, we demonstrated that PBT2 induced a translocation of zinc *in vitro* that increased the intracellular labile pool of zinc to result in a protein phosphatase-dependent (calcineurin) phosphorylation and inactivation of glycogen synthase kinase (GSK3) (Crouch *et al*., 2011). To determine whether this pathway was modulated in our *in vivo* model, we assessed protein levels of several protein phosphatases and their substrates in the hippocampus.

Calcineurin levels were unchanged as a function of PBT2 treatment in this model (data not shown). Consistent with this, there was no detectable change in the levels of GSK or phosphorylated GSK (data not shown). However, analysis of protein phosphatase 2A (PP2A), a serine/threonine protein phosphatase that has been implicated in a variety of cellular processes (Braithwaite *et al*., 2012), revealed that two of its subunits were significantly increased in the hippocampus following PBT2 treatment in aged mice [PP2A subunit A, unchanged; PP2A subunit B, PBT2 (+21%), *P* = 0.003; PP2A subunit C, PBT2 (+22%), *P* = 0.04; these subunits represent the scaffolding, regulatory and catalytic subunits of the PP2A heterotrimer, respectively] (Fig. 4c). As PP2A is implicated in the pathogenesis of AD and is the major phosphatase for the tau protein (Goedert *et al*., 1995), we assessed the levels of phosphorylated tau (Ser-396) and found that it was significantly reduced following PBT2 treatment (PBT2 (−81%), *P* = 0.03) (Fig. 4c). Thus, it is likely that the PP2A pathway is modulated by PBT2 and the reduction in phosphorylated tau, which we also observed in APP/PS1 mice following acute PBT2 treatment (Adlard *et al*., 2008), may contribute to the maintenance of normal anatomical and cognitive function. As PP2A is zinc sensitive (Xiong *et al*., 2013) and is known to be decreased during aging (Veeranna *et al*., 2011) (and also in AD), we hypothesize that a zinc deficit in the aged brain is overcome via the metal chaperone properties of PBT2, which subsequently results in an upregulation of PP2A. These data are consistent with our reported hypothesis that a characteristic of the 8-hydroxy quinolone family of metal chaperones is that they function to maintain cellular metal homeostasis. These compounds therefore are unlikely to have any effect in the absence of a perturbation in metal levels within the brain, as we have previously demonstrated in young wild-type animals dosed with PBT2 (Adlard *et al*., 2008).

## Discussion

A considerable global research effort has been dedicated to both elucidating and pharmacologically targeting the cellular and molecular processes involved in the learning and memory deficits that occur as a function of disease. In many cases, this cognitive dysfunction is the primary phenotypic feature that impacts the individual. While some psychiatric and neurological disorders, such as AD, have specific therapeutic targets, there are others that lack a definitive pathway that can be pharmacologically modulated in order to intervene in the progression of the clinical dysfunction. One such example is ARCD, which affects a significantly greater population than those with dementia alone and has yet to be effectively prevented by current therapeutics.

Age-related cognitive decline in a human population represents a complex phenomenon that has a heterogeneous aetiology. As such, there is unlikely to be a singular initiating cause for the erosion in learning and memory function that occurs. Mechanisms that have been put forward to account for cognitive loss with aging include inflammation and oxidative stress (Craft *et al*., 2012), alterations in brain neurochemistry/plasticity and connectivity (DeCarli *et al*., 2012) as well as epigenetic (Kosik *et al*., 2012) and other environmental/psychosocial factors (Kremen *et al*., 2012). While there are a multitude of pharmacological and nonpharmacological agents and therapies that have been proposed to intervene in many of these pathways in both health and disease (extensively reviewed in Husain & Mehta 2011), it is unlikely that there will be a ‘magic bullet’ that targets the entire spectrum of alterations that contribute to both brain and cognitive aging. In this study, however, we hypothesized that the broad biological effects of metal chaperones that we have characterized in transgenic mouse models of AD (Adlard *et al*., 2008, 2011) would be recapitulated in normal aged mice. Thus, we assessed the effect of PBT2 in preventing cognitive loss in aged C57Bl/6 mice and also characterized the effect of this metal chaperone on a number of anatomical and biochemical substrates that are critical to normal cognitive function and that have been postulated to contribute to ARCD.

Acute PBT2 treatment of aged mice resulted in a rapid improvement in performance in the Morris water maze, with treated animals both acquiring the task more rapidly and having better recall of the task than control-treated littermates. The performance of the PBT2-treated aged mice was indistinguishable from that of younger adult mice. The improvements were also seen after just 3 days of maze training (and after less than a week of drug treatment), similar to that which was shown in transgenic mouse models of AD that were treated with PBT2 using a similar protocol (Adlard *et al*., 2008). These data support the notion that PBT2 is able to rapidly improve spatial learning and memory in aged wild-type rodents. It is important to note, however, that PBT2 is unlikely to be a generic cognitive enhancer, as it does not alter water maze performance in young cognitively normal rodents (Adlard *et al*., 2008). This suggests that PBT2 targets deficits in one or more pathways relevant to cognition, and also allays the potential safety concerns that arise when considering cognitive enhancers that are effective in ‘normal’ individuals. In order to further define the underlying mechanisms of this improved cognition, we examined several anatomical and biochemical substrates of cognition.

As discussed within the results section, there was a breadth of biological effects within the brain following PBT2 treatment in the aged mice. While it is not possible to discern which of these was the principal driver of the cognitive benefit observed, it is likely to have resulted from a PBT2-mediated improvement in the function of different cellular pathways (particularly involving glutamatergic signalling pathways at the synapse) that are critical to synaptic plasticity and cognitive function (evidenced by increases in proteins such as CamKII, CREB, AMPA, NMDA and VGLUT1 as well as subregion-specific increases in zinc and glutamate within the hippocampus). In the longer term, these improvements are likely to synergize with the effects observed on neuronal health and connectivity (evidenced by increased neuron numbers, dendritic spines and synaptophysin content and perhaps contributed to in part by an action on neurogenesis within the brain) to foster a long-term improvement in both brain and cognitive health. As deficits in many of these same pathways are implicated in a variety of disorders, this also establishes a landscape where PBT2 may be efficacious in the treatment of a broad spectrum of diseases. Further to this, we have also identified a new *in vivo* target of PBT2, PP2A, which is involved in the pathogenesis of neurodegenerative diseases (including AD and other tauopathies) and which is considered to be a major therapeutic target to limit the pathological accumulation of the tau protein.

While there remain other mechanistic possibilities to account for the PBT2-mediated benefits observed in this study, it is clear that this compound has a broad suite of effects within the brain that translate to improved cognitive function in animal models of aging and disease (Adlard *et al*., 2008). That this activity has also translated to improved cognition in a short-term phase IIa human clinical trial of AD (Lannfelt *et al*., 2008; Faux *et al*., 2010) provides strong support for the efficacy of this compound in restoring normal brain function. The use of metal chaperones, such as PBT2, as novel therapeutic compounds for the treatment of both ‘normal’ and ‘pathological’ cognitive decline is strongly endorsed by these findings and warrants further mechanistic investigation into the precise mechanism of action of this class of compound, as well as human clinical trials to validate these rodent data.

## Experimental procedures

### Ethics statement

All animal experiments were approved by the Howard Florey Animal Ethics Committee and were conducted in accordance with the Australian Code of Practice for the Care and Use of Animals for Scientific Purposes as described by the National Health and Medical Research Council of Australia.

### Compound

PBT2 was provided by Prana Biotechnology. The chemical structure of PBT2 has previously been published (Telpoukhovskaia & Orvig, 2013).

### Treatment regime

Animals (female C57Bl/6 mice, purchased at 8 months of age from the Animal Resources Centre, Western Australia, and then aged in our Animal Facility) were randomly assigned to receive either standard suspension vehicle (SSV; 0.9% NaCl, 0.5% Na-carboxymethylcellulose, 0.5% benzyl alcohol and 0.4% Tween-80) (22 months old; *n* = 21), drug (22 months old; PBT2, 30 mg kg^−1^; *n* = 23) or SSV (12 months old; *n* = 7) once daily by oral gavage. These animals and the associated harvested tissues were used for all experiments in this study with the exception of the open field, rotarod and pole test behavioural experiments. A separate cohort of mice (22 months old; *n* = 5 SSV, *n* = 5 PBT2) was used for these three behavioural endpoints so that they could be conducted at the end of the short treatment regime without interfering in the primary endpoint of the study, the Morris water maze and all other histological and biochemical studies.

Animals were dosed in the afternoon for 11 consecutive days (to avoid any confounding issues that may arise from gavaging prior to behavioural studies), and then on the final 12th day of the study, animals were given a single dose of either SSV or drug 1 h prior to culling in the morning.

Experiments were profiled in a blinded manner to reduce any experimental bias. To achieve this, animals were gavaged by a technician who was not involved in any subsequent aspects of the studies. For all behavioural studies, animals were coded by a researcher not involved in the collection or analysis of the data, which was then done in a blinded manner. Likewise, all tissue samples were assigned a separate numerical code for all subsequent studies, and experimental data were generated and analysed based on this coding.

### Behavioural analyses

The Morris water maze was used to assess spatial learning and memory, as previously reported (Adlard *et al*., 2008). The acclimation to the water maze was performed on day 4 of dosing, then the learning trials were conducted on dosing days 5–10, then the probe trial was conducted on dosing day 11, followed by culling on dosing day 12. The pole test, rotarod and open field were used to assess motor function in a separate group of mice and were performed as previously described (Lei *et al*., 2012). However, one minor modification to the rotarod analysis was made, in that we examined both the latency to fall (as described in Lei *et al*., 2012) and the survival on the rod (i.e. the percentage of animals that were able to stay on the rod with each increase in speed level). The open field test was performed on day 7 of dosing, the pole test on day 9 of dosing and the rotarod test on day 11 of dosing.

### Golgi

We utilized Golgi analysis, as previously reported (Adlard *et al*., 2011). Animals were deeply anaesthetized with sodium pentobarbitone (100 mg kg^−1^) and then transcardially perfused with 0.01 m ice-cold PBS. Brains were then rapidly removed and cut into 4-mm blocks. The blocks containing the hippocampus were incubated in rapid golgi solutions (Rapid Golgi Stain Kit; FD Rapid GolgiStain kit, FD NeuroTechnologies, Inc., Columbia, MD, USA) according to the manufacturer’s instructions. The blocks were infused with a solution containing potassium dichromate, potassium chromate and mercuric chloride for 2 weeks. The blocks were then snap-frozen and cut at 90 μm using a microtome (Cryostat, Leica CM 18–50; Leica Microsystems GmbH, Wetzlar, Germany). Sections were collected at the level of the hippocampus (bregma −1.40 to −2.70) and thaw-mounted onto gelatinized microscope slides. The slides were dried, dehydrated, cleared with xylene and mounted with distyrene-plasticized xylene. Golgi-treated brain tissue was analysed using a light microscope with an oil immersion lens [63×, NA 1.3; Leica DM4000B; Leica Microsystems; with a further magnification from the projection lens to the camera (10×) used for all measurements, providing a total magnification of 630 times]. The whole neuron was traced manually.

Five neurons from each animal were selected for hippocampal spine analysis. Neurons were selected only if they were clearly identified as being from the CA1 subfield, they appeared completely filled and they were far enough from the neighbouring Golgi-stained cells to be individually identified. Tertiary or greater order apical and basal dendrites (*n* = 5 apical and *n* = 5 basal) were selected and analysed to determine spine density, dendritic length and dendritic number. Thus, 50 dendrites were sampled from each animal. The standard deviations indicate the uniformity of the analyses and demonstrate that this is a representative sampling.

### Histology

Mice were perfused with PBS (pH 7.4), and the brain was transferred into 4% paraformaldehyde overnight and then transferred to 30% sucrose in PBS, prior to being snap-frozen and cut (30 μm, 1:4) onto gelatin-coated slides. The slides were postfixed with 4% paraformaldehyde for 1 min, washed with PBS, blocked in 10% normal goat serum [0.1 m phosphate buffer (PB), 30 min] and then incubated with primary antibody (doublecortin (DCX), 1:300, Cell Signaling Technology (Danvers, MA, USA); Ki-67, 1:300, Abcam; NeuN (Cambridge Science Park, Cambridge, UK), 1:1500, Merck, White House Station, NJ, USA). After an overnight incubation, the slides were washed with PBS and incubated for 3 h at room temperature with either goat anti-mouse or goat anti-rabbit IgG poly-HRP (Millipore, Billerica, MA, USA) 1:2 diluted in 0.1 m PB. The slides were incubated in a nickel-3,3‘-diaminobenzidine (DAB) solution containing 0.05% DAB, 0.05% nickel chloride and 0.05% cobalt chloride and further developed by adding 0.001% hydrogen peroxide for 5 min. Sections were then washed, dehydrated and mounted. DCX- and Ki-67-positive cells were counted in the hippocampus (four sections per series per mouse) using a 63× oil immersion objective. Stereological analysis (Stereo Investigator) was used to estimate the total number of hippocampal neurons (NeuN-positive cells). The cells were counted using different counting grids (*x*, *y*) and counting frames according to the region: for DG and area CA2, 100 × 100 μm and 12 × 12 μm; for area CA1, 150 × 150 μm and 14 × 14 μm; and for area CA3, 150 × 150 μm and 23.5 × 23.5 μm.

### Western blot

Hippocampi were homogenized in 15 volumes of ice-cold PBS containing Complete Protease Inhibitor Cocktail Tablets (Roche Applied Science, Indianapolis, IN, USA) and Phosphatase Inhibitor Cocktail 1 and 2 (Sigma-Aldrich, St. Louis, MO, USA). Samples were prepared for PAGE by the addition of 4× NuPAGE lithium dodecyl sulfate sample buffer and 10× NuPAGE sample reducing agent (to a final 1× concentration). Samples were heated to 70 °C for 10 min, loaded onto NuPage Novex 4–12% Bis-Tris gels and run at 130 V for 90 min. Gels were transferred to nitrocellulose using the iBlot Gel Transfer Device (Life Technologies, Grand Island, NY, USA) set to program 3. Membranes were heated for 5 min in PBS, blocked in tris-buffered saline with tween 20 (TBST) containing 5% skim milk powder and then incubated with primary antibody overnight at 4 °C. Blots were rinsed in TBST and incubated with secondary antibody (1 h, RT), followed by further rinsing, development with enhanced chemiluminescence reagent and imaging using a Fujifilm Luminescent Image Analyser LAS-3000 and Image Reader LAS-3000 software package (R&D Systems, Minneapolis, MN, USA). Data were normalized to total protein loaded and to the GAPDH loading control.

### Laser ablation inductively coupled plasma mass spectrometry

Laser ablation inductively coupled plasma mass spectrometry imaging and quantitation was performed as previously described (Hare *et al*., 2010).

### Glutamate

The Glutamine and Glutamate Determination Kit (Sigma-Aldrich) was used to assess total glutamate levels in hippocampal tissue. The manufacturer’s recommended protocol was followed.

### Statistical analysis

Statistical analysis was performed using GraphPad Prism (version 5.0f, GraphPad Software, Inc., La Jolla, CA, USA). Normality was assessed using D’Agostino-Pearson omnibus K2 test. In cases of equal sample variance, *t-*tests were performed (two-tailed), and where variances between groups were significantly different, a Welch correction was applied. For water maze analysis, a repeated-measures ANOVA and *post hoc t*-tests (including Tukey’s multiple comparison corrections) were utilized. Significance was set at *P* = 0.05.

## Supporting Information

**Fig. S1** Concentrations of metals within different regions of the mouse brain, assessed by laser ablation ICPMS.
