# Supplementary figures and images for: A novel approach to rapidly prevent age-related cognitive decline
Source: Aging Cell. 2013 Dec 4;13(2):351–9. doi: 10.1111/acel.12178 (PMC4331782; doi:10.1111/acel.12178)

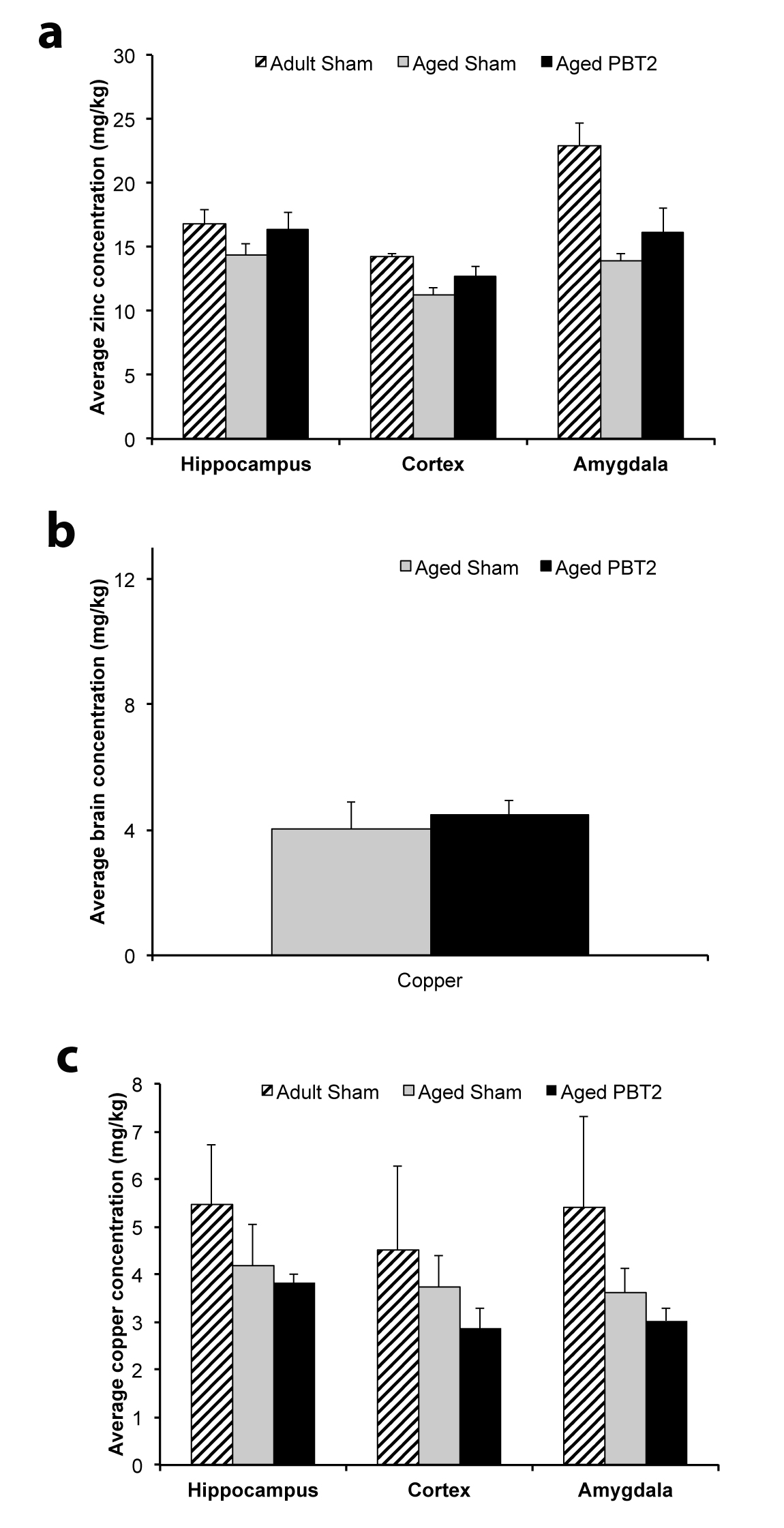

Supplement: Supplementary file 1 — Fig. S1 Concentrations of metals within different regions of the mouse brain, assessed by laser ablation ICPMS. [file acel0013-0351-sd1.jpg]
